# TIE2-expressing monocytes and M2-polarized macrophages impact survival and correlate with angiogenesis in adenocarcinoma of the pancreas

**DOI:** 10.18632/oncotarget.25690

**Published:** 2018-07-03

**Authors:** Georgi Atanasov, Charlotte Pötner, Gabriela Aust, Katrin Schierle, Corinna Dietel, Christian Benzing, Felix Krenzien, Michael Bartels, Uwe Eichfeld, Moritz Schmelzle, Marcus Bahra, Andreas Pascher, Georg Wiltberger

**Affiliations:** ^1^ Department of Visceral, Transplantation, Thoracic and Vascular Surgery, University Hospital Leipzig, Leipzig, Germany; ^2^ Department of Surgery, Campus Charité Mitte und Campus Virchow-Klinikum, Charité, Universitätsmedizin Berlin, Berlin, Germany; ^3^ Berlin Institute of Health, Berlin, Germany; ^4^ Department of Surgery, Research Laboratories, University of Leipzig, Leipzig, Germany; ^5^ Institute of Pathology, University Hospital Leipzig, Leipzig, Germany; ^6^ Department of General and Visceral Surgery, Helios Clinic Leipzig, Leipzig, Germany; ^7^ Department of General, Visceral and Transplantation Surgery, University Hospital of RWTH Aachen, Aachen, Germany

**Keywords:** TIE2-expressing monocytes, pancreatic adenocarcinoma, tumor-associated macrophages, M2-macrophages, angiogenesis

## Abstract

**Introduction:**

M2-polarized tumor-associated macrophages (TAMs) and TIE2-expressing monocytes (TEMs) are associated with angiogenesis and have been identified as a potential prognostic marker in several solid tumors, including hepatobiliary malignancies. However, little is known regarding their influence on tumor progression and patient survival in pancreatic ductal adenocarcinoma (PDAC).

**Results:**

Patients with tumors characterized by the presence of CD163^+^ TAMs or TEMs in TCA or TIF, respectively, showed a significantly decreased 1-, 3- and 5-year overall and recurrence-free survival compared to patients without CD163^+^ TAMs or TEMs (all *ρ* < 0.05). Patients with TEMs in TCA showed a higher incidence of tumor recurrence (*ρ* < 0.05). Furthermore, the presence of CD163^+^ TAMs was associated with a higher tumor MVD (*ρ* < 0.05).

**Conclusions:**

Presence of M2-polarized TAMs and TEMs is associated with a decreased overall and recurrence-free survival of patients with PDAC.

**Materials and methods:**

The localization and density of CD163^+^ M2-polarized TAMs and TEMs were quantified in the tumor central area (TCA) and tumor-infiltrating front (TIF) in human PDAC tissue (*n* = 106) and correlated to clinicopathological characteristics, tumor recurrence rates and patient survival. In parallel, tumor microvascular density (MVD) and the density of angiopoietin-positive tumor cells were quantified. Statistical analysis was performed using SPSS software.

## INTRODUCTION

Solid cancers harbor different subpopulations of immune cells, each of which has the potential for pro- or anti-tumor functions. Macrophages are the predominant immune cell population found throughout tumors and macrophage numbers or functions can be modulated in order to augment anti-cancer therapies [1]. The immune activation state of these tumor-associated macrophages (TAMs) can be divided into M1- (anti-cancer function) and M2-type (pro-tumoral or immunosuppressive) [2]. Various escape mechanisms deployed by malignant cells and the tumor microenvironment (TME) mediate the conversion of infiltrating macrophages into immunosuppressive and pro-tumoral M2 TAMs [3]. Therefore, understanding and targeting the specific pathways associated with the M2 polarization state of TAMs is a promising approach for therapeutic strategies.

As previously reported by us, a differential influence and functionality of localized tumor sites in TME, i.e. tumor central area (TCA) and tumor invasive front (TIF), on related immunologic responses play a key role in cancer immunity and tumor progression [4]. TAMs localized in TCA or TIF can impact oncogenesis in an opposite manner. High TAMs frequency in TCA is associated with poor prognosis in hepatocellular carcinoma (HCC), esophageal, ovarian and breast cancer [5–10]. By contrast, TAMs in TIF in colonic, gastric, and endometrial cancer correlate with a favorable outcome [11–13]. TME, tumor-derived factors and escape mechanisms facilitate tumor growth, neovascularization, and metastasis. However, a constant interplay between immune responses in TCA or TIF and infiltrating TAMs and their polarization states, plays a key role in this process.

Tumor angiogenesis is a major factor in the development and progression of cancer and therefore inhibiting it is a promising therapeutic target [14]. Members of the angiopoietin (Ang) family are potent growth factors and important modulators of tumor angiogenesis but could represent functional antagonists as well [15]. Monocytes/macrophages are primary producers of angiogenic mediators in oncogenesis [16–17]. Bone marrow (BM)-derived myeloid cells contribute significantly to tumor angiogenesis [18]. In both mice and humans, a subset of proangiogenic circulating and tumor-infiltrating monocytes expressing functionally active Ang receptor TIE2 have been recently identified [19]. These Tie2-expressing monocytes (TEMs) are selectively recruited to tumors, foster neoangiogenesis, and oncogenesis, but are not found in non-cancerous tissues [20, 21]. We have previously demonstrated that in respect to tumor vascularization grade, monocyte localization in TCA or TIF and related cancer immunity, these tumor-infiltrating TEMs and TAMs can impact survival and prognosis in an opposite manner [4, 22, 23].

Clinical significance of angiogenic TEMs and M2-polarization state of infiltrating TAMs has been suggested for human malignancies, where the interplay between tumor immunology and angiogenesis plays a key role in oncogenesis [20]. Recently, we showed that tumor Ang density, corresponding angiogenic TEMs and infiltrating TAMs impact patent survival and exert prognostic significance in primary hepatic tumors and hilar cholangiocarcinomas [4, 22, 23]. However, the importance of these immune markers in pancreatic ductal adenocarcinoma (PDAC) is unknown. The aim of this study was therefore to evaluate the importance of M2-polarized TAMs, Angs and related Ang-receptor bearing TEMs and their association with tumor angiogenesis, tumor growth, metastasis, recurrence and clinical prognosis in human PDAC.

## RESULTS

The overall 1-, 3- and 5-year survival of our cohort was 81.2, 44.9 and 41.8%, respectively. The recurrence-free 1-, 3- and 5- year survival rates were 75.7, 45.1 and 43.9%, respectively. Sixty-four out of the 106 (64/106, 60.4%) patients died within the follow-up interval. In 52/106 patients (49.1%) tumor recurrence was diagnosed and 40/106 (37.7%) patients died of recurrent disease. Local tumor recurrence was detected in 20/106 (18.9%) patients. In 50/106 (45.9%) patients distant metastases were seen (Table 1). 15/106 (14.1%) patients had a local recurrence and metastatic disease. Nineteen/106 (17.9%) patients died from other causes and had no signs of tumor recurrence at the time of death.

**Table 1:** Clinicopathological characteristics of the patients included in the study

Clinicopathological characteristics	
Variable	Value (%)
No. of patients	106
Gender	
Female	41 (38.7%)
Male	65 (61.3%)
Patient age	
≤60	55 (51.9%)
>60	51 (48.1%)
Pathologic T stage	
T1/T2	22 (20.8%)
T3/T4	84 (79.2%)
Pathologic N stage	
Positive	32 (30.2%)
Negative	74 (69.8%)
Lymphangiosis carcinomatosa	
Positive	23 (21.7%)
Negative	83 (78.3%)
Angionvasion	
Positive	29 (74.5%)
Negative	27 (25.5%)
Perineural sheath infiltration	
Positive	35 (33.0%)
Negative	71 (67.0%)
Pathologic R-Category	
R0	82 (77.4%)
R1/R2	24 (22.6%)
Histologic differentiation	
Well	4 (3.8%)
Moderate/Poor	102 (96.2%)
Distant metastases	
With	47 (44.3%)
Without	59 (55.7%)
Liver metastases	
With	27 (25.5%)
Without	79 (74.5%)
Tumor recurrence	
With	52 (49.1%)
Without	54 (50.9%)
Local recurrence	
With	20 (18.9%)
Without	86 (81.1%)

### TEMs, TAMs and angiopoietin distribution in PDAC

**Figure 1:**
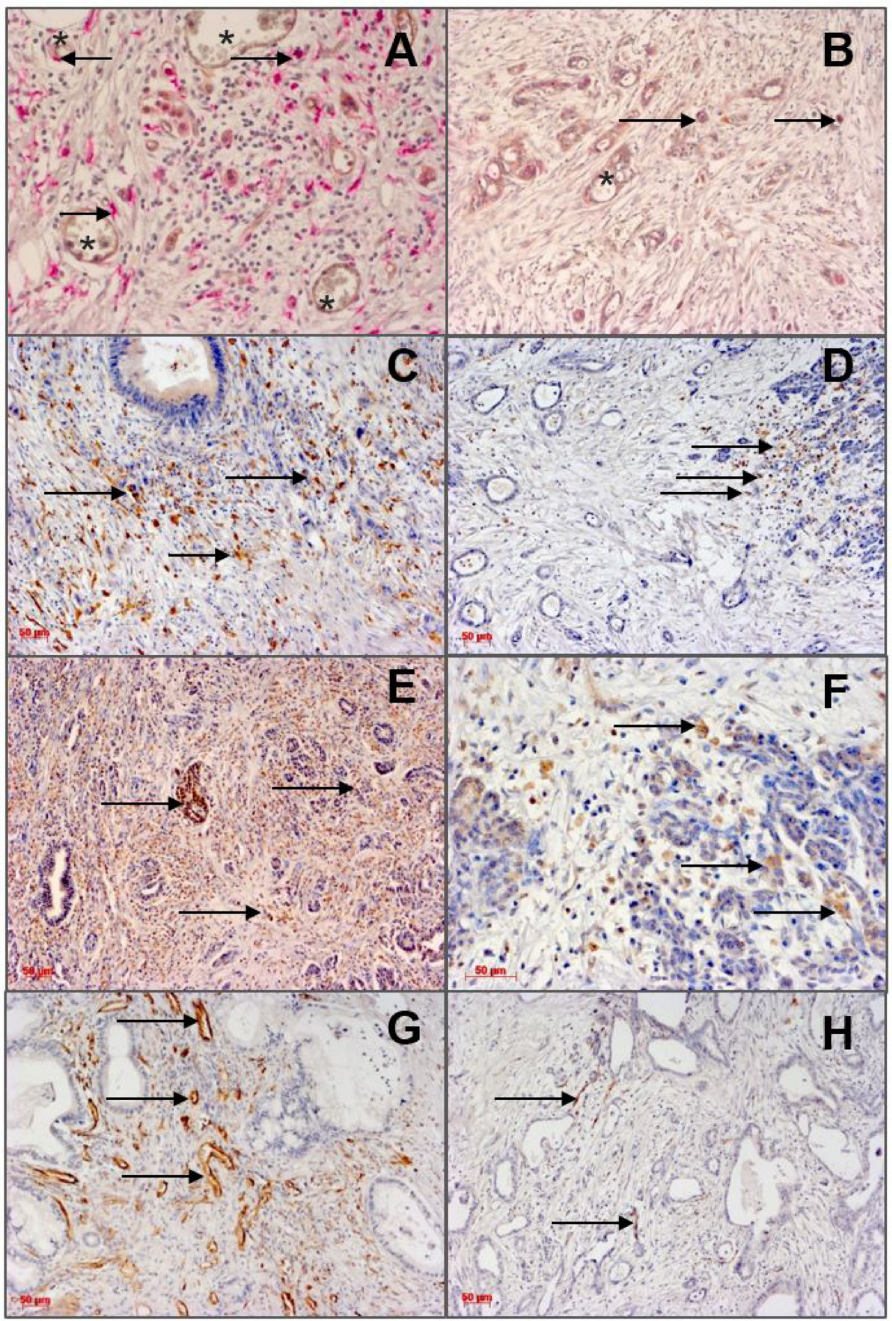
Immunohistological detection of angiopoietins and infiltrating monocytes/macrophages (left column: high density; right column: low density) in TCA of PDAC specimens Arrows indicate positive staining, asterisks indicate microvessels. Scale bar 50 µm. (**A**): High density of TIE2+ monocytes in the tumor central areas (TCA). PDAC revealed a homogenous infiltration pattern of these cells with preference for the perivascular areas. (**B**): Low density of TIE2+ monocytes (arrows). (**C**): High density of CD163+ TAMs. (**D**): Low density of CD163+ TAMs. (**E**) High Ang-2 expression. (**F**): Low Ang-2 expression. (**G**): CD31+- microvessels (high microvascular density, high MVD. (**H**): Low MVD.

Typical images for the tissue distribution of TEMs, CD68^+^ or CD163^+^ TAMs and angiopoietins are shown in Figure 1A–1H, the respective statistical evaluation of all patients is summarized in Tables 2, 3 and 4. TEMs are preferentially located in areas of tumor neovascularization, but not in tumor necrosis regions (Figure 1A–1B). CD68^+^ or CD163^+^ TAMs showed a uniform density in the tumor stroma, TCA, TIF and were also present in necrotic tumor regions (Figure 1C–1D). The pattern of TEMs and CD163^+^ TAMs displayed a preference for the tumor perivascular areas (Figure 1A). High Ang density (Figure 1E–1F) was not frequently observed in PDAC, as most cases showed Ang low abundance. In contrast, most of PDAC samples displayed high tumor microvascular density (MVD) (Figure 1G–1H).

**Table 2:** Correlation of TIE2-expressing monocytes in tumor central area with clinicopathological characteristics of pancreatic adenocarcinoma

Clinicopathological analysis			
Variable	TEMs pos	TEMs neg	*p*
No. of patients	7	99	
Gender			0.570
Female	2 (21.6%)	39 (39.4%)	
Male	5 (71.4%)	60 (60.6%)	
Patient age, years			0.621
>60	4 (57.1%)	47 (47.5%)	
≤60	3 (42.9%)	52 (52.2%)	
Pathologic T stage			0.662
T1/T2	1 (14.3%)	21 (21.2%)	
T3/T4	6 (85.7%)	78 (78.8%)	
Pathologic N stage			0.072
Positive	0 (00.0%)	32 (32.3%)	
Negative	7 (100.0%)	67 (67.7%)	
Lymphangiosis carcinomatosa			0.150
Positive	0 (00.0%)	23 (23.2%)	
Negative	7 (100.0%)	76 (76.8%)	
Angioinvasion			0.482
With	6 (85.7%)	73 (73.7%)	
Without	1 (14.3%)	26 (26.3%)	
Perineural sheath infiltration			0.796
Positive	2 (28.6%)	33 (33.3%)	
Negative	5 (71.4%)	66 (66.7%)	
Pathologic R-category			0.585
R0	6 (85.7%)	76 (76.8%)	
R1/R2	1 (14.3%)	23 (23.2%)	
Histologic differentiation			0.588
Well	0 (00.0%)	4 (4.0%)	
Moderate/poor	7 (100.0%)	95 (96.0%)	
Distant metastases			**0.023**
With	6 (85.7%)	41 (41.4%)	
Without	1 (14.3%)	58 (58.6%)	
Liver metastases			**0.047**
With	4 (57.1%)	23 (23.2%)	
Without	3 (42.9%)	76 (76.8%)	
Tumor recurrence			**0.045**
With	6 (85.7%)	46 (46.5%)	
Without	1 (14.3%)	53 (53.5%)	
Local recurrence			0.093
With	3 (42.9%)	17 (17.2%)	
Without	4 (57.1%)	82 (82.8%)	
MVD			0.255
High	6 (85.7%)	64 (64.6%)	
Low	1 (14.3%)	35 (35.4%)	
CD163-positive			**0.002**
TAMs in TIF			
Positive	1 (50.0%)	56 (36.0%)	
Negative	5 (50.0%)	17 (64.0%)	
CD68-positive			0.480
TAMs in TCA			
Positive	4 (57.1%)	43 (43.4%)	
Negative	3 (42.9%)	56 (56.6%)	

**Table 3:** Correlation of CD163-positive TAMs in the tumor invasive front with clinicopathological characteristics of pancreatic adenocarcinoma

Clinicopathological analysis			
Variable	CD163 pos	CD163 neg	*p*
No. of patients	23	59	
Gender			0.127
Female	12 (52.2%)	20 (33.9%)	
Male	11 (47.8%)	39 (66.1%)	
Patient age, years			0.817
>60	10 (43.5%)	24 (40.7%)	
≤60	13 (56.5%)	35 (59.3%)	
Pathologic T stage			0.254
T1/T2	6 (26.1%)	9 (15.3%)	
T3/T4	17 (73.9%)	50 (84.7%)	
Pathologic N stage			0.495
Positive	6 (26.1%)	20 (33.9%)	
Negative	17 (73.9%)	39 (66.1%)	
Lymphangiosis carcinomatosa			0.284
Positive	3 (13.0%)	14 (23.7%)	
Negative	20 (87.0%)	45 (76.3%)	
Angioinvasion			0.123
With	21 (91.3%)	45 (76.3%)	
Without	2 (8.7%)	14 (23.7%)	
Perineural sheath infiltration			0.178
Positive	5 (21.7%)	22 (37.3%)	
Negative	18 (78.3%)	37 (62.7%)	
Pathologic R-category			0.641
R0	19 (82.6%)	46 (78.0%)	
R1/R2	4 (17.4%)	13 (22.0%)	
Histologic differentiation			0.271
Well	0 (0.0%)	3 (5.1%)	
Moderate/poor	23 (100.0%)	56 (94.9%)	
Distant metastases			0.074
With	14 (60.9%)	23 (39.0%)	
Without	9 (39.1%)	36 (61.0%)	
Liver metastases			0.052
With	9 (39.1%)	11 (18.6%)	
Without	14 (60.9%)	48 (81.4%)	
Tumor recurrence			0.085
With	15 (65.2%)	26 (44.1%)	
Without	8 (34.8%)	33 (55.9%)	
Local recurrence			0.977
With	5 (21.7%)	13 (22.0%)	
Without	18 (78.3%)	46 (78.0%)	
MVD			**0.046**
High	4 (17.4%)	24 (40.7%)	
Low	19 (82.6%)	35 (59.3%)	
CD68-positive			0.480
TAMs in TCA			
Positive	15 (65.2%)	41 (71.9%)	
Negative	8 (34.8%)	16 (28.1%)	

**Table 4:** Patient groups and scoring in patients with pancreatic adenocarcinoma (*n* = 106)

Antigen(s)	Use	Tumor area	Groups/number of patients	
angiopoetin-1	determination of angiopoetin-1 in tumor cells	TCA	ANG1^high^ score 1: positive *n* = 39	ANG1^low^ score 0: negative *n* = 67
angiopoetin-2	determination of angiopoetin-2 in tumor cells	TCA	ANG2^high^ score 1: positive *n* = 26	ANG2^low^ score 0: negative *n* = 80
CD14, TIE2	determination of TEMs	TCA	positive/presence of TEMs (in short: TEM^+^) *n* = 99	negative/absence of TEMs (in short: TEM^−^) *n* = 7
CD31	determination of tumor MVD	area of highest MVD, “vascular hot spot”	MVD^high^ (≥50 microvessels/10 optical fields) *n* = 69	MVD^low^ (<50 microvessels/10 optical fields) *n* = 37
CD68	determination of TAMs	TIF	positive/presence of CD68^+^ TAMs (in short: CD68^+^ TAM^+^) *n* = 73	negative/absence of CD68^+^ TAMs (in short: CD68^+^ TAM^−^) *n* = 9
CD163	M2-polaryzation of TAMs	TIF	positive/presence of CD163^+^ TAMs (in short: CD163^+^ TAM^+^) *n* = 23	negative/absence of CD163^+^ TAMs (in short: CD163^+^ TAM^−^) *n* = 59

Abbreviations: MVD: microvascular density; TAM: tumor associated macrophage; TCA: tumor central area; TEM: Tie2-expressing monocyte; TIF: tumor infiltrating front.

### TEMs associate with metastatic disease and tumor recurrence in PDAC

The presence of TEMs in the TCA was associated with an enhanced incidence of metastatic disease (Figure 1A–1B, Tables 2 and 4). In the TEM^+^ group 6/7 (85.7%) and in the TEM^-^ group 41/99 (41.4%) patients had distant metastases (*ρ* = 0.023). Interestingly, a significant association between infiltrating TEMs and presence of hepatic metastases was observed: 76/99 (76.8%) patients of the TEM^−^ group had no distant metastases in the liver (*ρ* = 0.047). The presence of TEMs did not correlate to other clinicopathologic parameters or to Ang expression in PDAC. The frequency of CD68^+^ TAMs in TCA or TIF was not associated with the studied parameters.

### M2-polarized TAMs associate with enhanced tumor neovascularization in PDAC

The presence of CD163^+^ TAMs in TIF was associated with the MVD (Tables 3 and 4). In the CD163^+^ TAM^+^ group 19/23 (82.6%) patients, whereas in the CD163^+^ TAM^–^ group only 24/59 (47.0%) patients showed MVD^high^ (*ρ* = 0.046). Interestingly, TEMs in TCA and CD163^+^ TAMs in TIF were strongly associated: in the TIF CD163^+^ TAM^−^ group 56/59 (98.2%) patients were TEM^-^, whereas 17/23 (77.3%) patients in the TIF CD163^+^ TAM^+^ group were TEM^+^ in the TCA (*ρ* = 0.002).

### Angiopoietin-2 expression associates with perineural sheet infiltration in PDAC

Ang-2 expression in TCA correlated with the incidence of perineural sheet infiltration (*ρ* = 0.002) (Figure 1E–1F, Table 4). In the ANG2^low^ group, only 20/80 (25.0%) patients showed a perineural sheet infiltration, whereas in the ANG2^high^ group 11/26 (42.3%) patients had this phenomenon. Ang-1 expression in TCA was associated inversely with perineural sheet infiltration (*ρ* = 0.038): in the ANG1^high^ group, 31/39 (79.5%) had no perineural sheet infiltration.

### Influence of TIE2-expressing monocytes and M2-polarized macrophages on survival in PDAC

**Figure 2:**
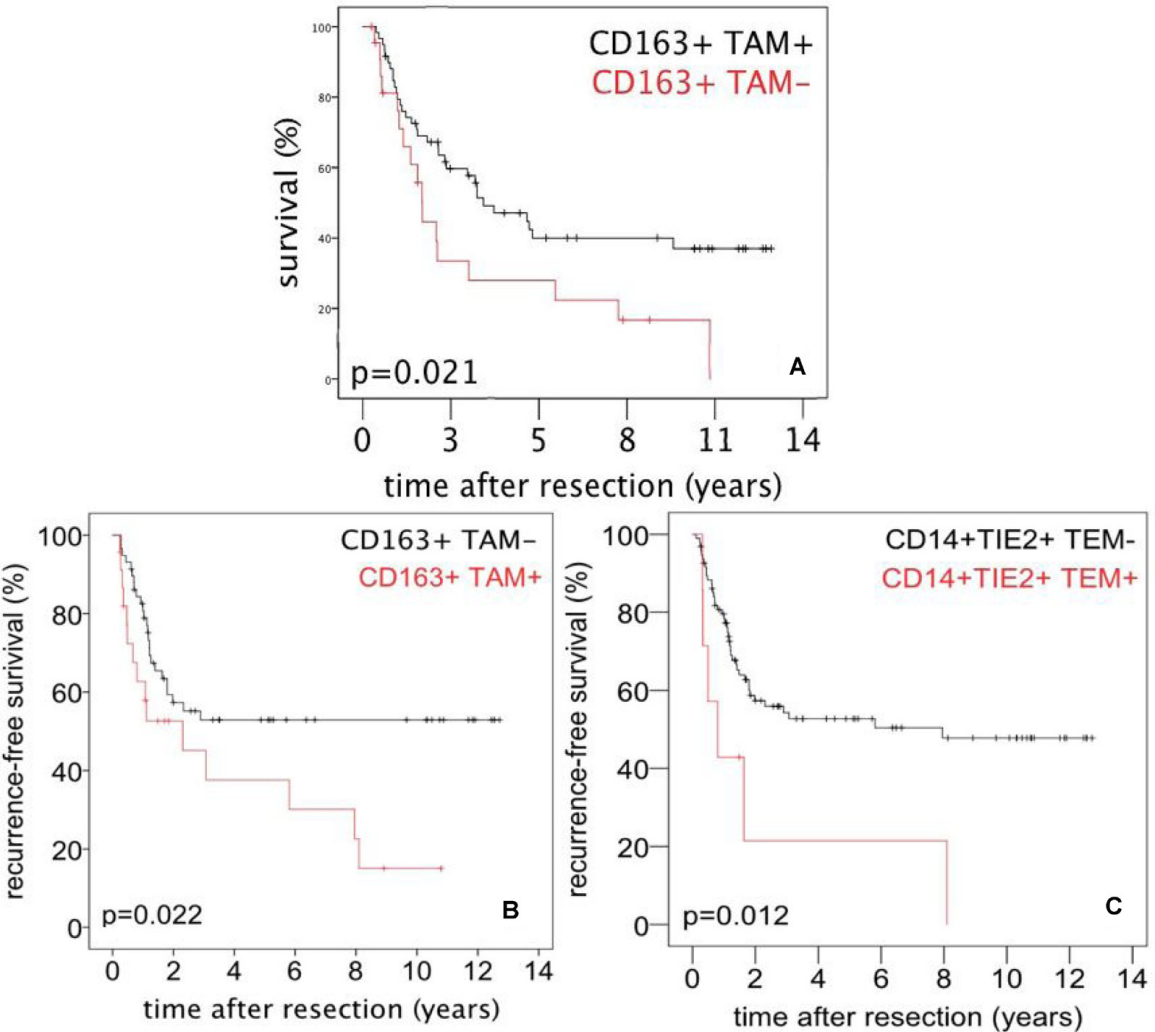
Survival after surgery for PDAC referred to tumor infiltrating monocytes/macrophages (**A**) Overall survival after surgery for PDAC referred to presence or absence of CD163^+^ TAMs in the tumor infiltration front (TIF). (**B**) Recurrence-free survival after surgery for PDAC referred to presence or absence of CD163^+^ TAMs in TIF. (**C**) Recurrence-free survival after surgery for PDAC referred to presence or absence of TEMs in the tumor central area (TCA).

Patient survival was decreased in patients with CD163^+^ TAMs in TCA (*p* = 0.041) or TIF (*p* = 0.021) when compared to patients without these cells in both tumor sites. The overall 1-, 3- and 5-year survival rates were 100, 84.3 and 84.3% in patients of the CD163^+^ TAM^-^ group compared to 76.2, 45.1 and 39.9% in the CD163^+^ TAM^+^ group, respectively, when considering the TIF (Figure 2A) or TCA (data not shown). Furthermore, the absence of CD163^+^ TAMs in TIF correlated with an improved recurrence-free survival. The 1-, 3-, and 5-year recurrence-free survival of patients without CD163^+^ TAMs were higher (82.4, 53.2, and 53.2%) compared to patients with CD163^+^ TAMs (58.1, 43.7, and 37.6%), respectively (*p* = 0.022; Figure 2B).

The 1-, 3-, and 5-year recurrence-free survival of patients without TEMs in TCA (77.6, 51.8 and 49.9%) were higher compared to patients with TEMs (42.9, 21.7 and 21.7%), respectively (*p* = 0.012; Figure 2C). The presence of CD68^+^ TAMs was not associated with patient survival (data not shown).

## DISCUSSION

Analyzing tissue density of infiltrating M2-polarized TAMs, TEMs, and Angs in tumor samples from patients who underwent resection for PDAC, we demonstrated that (1.) Ang density associates with perineural sheet infiltration (2.) angiogenic TEMs and M2-polarized TAMs are differentially expressed in PDAC, (3.) correlate with metastatic spread and tumor recurrence, (4.) associate with angiogenesis, and (5.) impact survival rates.

The mechanisms that enable solid cancers to escape elimination by the immune system remain unclear, but their elucidation may provide novel therapeutic interventions. Host immune function in TME, i.e. TCA and TIF, is shaped by tissue-specific and tumor-derived signals that reduce the potency of anti-tumor immune competence. This is particularly relevant in human oncogenesis where immune tumor infiltration with macrophage/monocyte subsets heavily affects prognosis [24]. M2 TAMs and TEMs express functional receptors of angiogenesis, build up to 50% of the tumor volume, exert a significant impact on cancer-related inflammation and angiogenesis, inhibit anti-tumor response, and facilitate tumor escape mechanisms, metastasis and tumor progression [25].

There is a scarce amount of data on the presence of TEMs and their correlation with clinicopathologic characteristics in human tumors. Furthermore, the significance of TEMs to date is established mainly regarding their circulating population in human blood [26, 27]. The few published studies failed to demonstrate a prognostic value of infiltrating TEMs in solid cancerous tissue. To our knowledge, the presented work is the first to document TEMs presence and importance in PDAC. Intratumoral visualization of TEMs using immunoreactivity revealed abundance in TCA and TIF, and a preferential localization in proximity to microvasculature, as well. Of note, as reported by us previously for other human cancers, a considerable amount of PDAC samples exhibited an absence of TEMs in solid cancerous tissue [23].

In our work presence of TEMs and M2 TAMs impacted patient survival after resection of PDAC. Multivariate analysis showed that tumor recurrence is an independent predictive factor for survival in patients with resectable PDAC (data not shown). Therefore, the positive correlation between infiltrating TEMs and recurrent disease supports the notion that TEMs may be a prognostic marker in patients with PDAC. In support of this, we found that patients with a higher TEMs frequency are at a greater risk of developing metastatic disease and especially liver metastases. Of note, the latter usually precludes curative surgery and in these cases patients are treated with systemic therapy for disease control. Taken together, these data implicate the potential use of TEMs and CD163^+^ TAMs as possible biomarkers in PDAC that could help deliver more individualized diagnostic and therapeutic modalities.

Members of the Ang family of angiogenic growth factors are potent regulators of blood- and lymphangiogenesis [28]. In agreement with previous reports, delineating a negative impact of Ang-2 in PDAC, in the current work Ang-2 frequency associated with increased incidence of perineural sheet infiltration [29]. However, we have previously shown Ang-1 to be associated beneficial tumor characteristics in other hepatobiliary malignancies [23]. In line with this, in the current work Ang-1 distribution revealed an inverse correlation with incidence of perineural sheet infiltration. Taken together our data is in concordance with published results indicating functional antagonism within the human angiopoietin axis.

The descriptive nature of the presented results represents a limitation of the current work. Thus, functional tests, such as *in vitro* co-culturing of TEMs and tumor-derived factors/tumor cells to measure and verify M2-related phenotype and responses will allow a greater understanding of this proposed interaction. This could help deliver novel strategies for adjunct treatments to augment the efficacy of checkpoint blockade inhibitors or more standard chemotherapies, radiation, and surgery.

In summary, in the current work M2-polarized TAMs, related angiopoietin-axis and corresponding Ang-receptor bearing TEMs associated with established clinicopathologic characteristics and survival in human PDAC. Consequently, further research is required to investigate molecular mechanisms linking cancer immunity to TEMs functions, phenotype and polarization state.

## MATERIALS AND METHODS

### Patients and tumor samples

A total of 106 patients who underwent major pancreatectomy for pancreatic adenocarcinoma at the Department of Visceral-, Transplantation-, Thoracic- and Vascular Surgery, University Hospital Leipzig, Leipzig, Germany were included in the study. Adenocarcinoma of the pancreas was confirmed histopathologically and classified according to the Union for International Cancer Control (UICC) classification. This study was carried out in accordance with the recommendations of the Ethics Committee of the Medical Faculty of the Leipzig University (no. 234-14-14072014).

In all patients, pancreas resection was with curative intent and none of the patients received neoadjuvant radio- and/or chemotherapy prior to surgery. None of the patients died in the postoperative period. Tissue blocks embedding a representative sample of the tumor were retrieved from the files of the Institute of Pathology. Histological diagnosis of the primary tumor stage and nodal status were determined by hematoxylin and eosin (H&E) stained sections. Histological evaluation of all specimens was performed by two investigators (CP and GA) with formal training in histopathology, and an independent pathologist (KS), without any knowledge of prognosis or clinicopathological variables. Paraffin-embedded, formalin-fixed tumor samples were used.

### Immunohistology

Protocols for immunohistology and quantification of cellular infiltrates in paraffin-embedded, formalin-fixed tissue samples have been published previously [4, 22, 23]. Table 5 summarizes the antibodies and staining conditions used. Briefly, 5 µm tumor sections were dewaxed and rehydrated. After antigen retrieval and blocking of the endogenous peroxidase or alkaline phosphatase activity, the sections were consecutively incubated with the primary and enzyme-labeled secondary antibody and the respective enzymatic substrate. Sections were counterstained with hematoxylin. Specificity controls were performed without the primary antibody.

**Table 5:** Antibodies used for immunostaining

Antigen	m/p	clone	species	company	secondary antibody	company	substrate	antigen retrieval
angiopoetin-1	p	−	goat	Santa Cruz, Dallas, USA	rabbit anti-goat-Ig	Agilent	DAB	10 mM citrate, pH 5.5
angiopoetin-2	p	−	goat	Santa Cruz, Dallas, USA	rabbit anti-goat-Ig	Agilent	DAB	10 mM citrate, pH 5.5
CD14	p	−	rabbit	Sigma Aldrich, Steinheim, Germany	anti-rabbit Ig/AP	Vector Laboratorie, Burlingame, USA	Vector Red AP	target retrieval solution pH 6.1
CD31	m	JC70A	mouse	Agilent Technologies Deutschland GmbH, Waldbronn, Germany	anti-mouse-Ig/ peroxidase	Vector	DAB	Tris/EDTA pH 9.0
CD68	m	PG-M1	mouse	Agilent	anti-mouse-Ig/ peroxidase	Vector	DAB	Tris/EDTA pH 9.0
CD163	m	10D6	mouse	Leica Biosystems, Newcastle Upon Tyne, UK	anti-mouse-Ig/ peroxidase	Vector	DAB	10 mM citrate, pH 5.5
TIE2	p	−	goat	R&D Sys, Minneapolis, USA	anti-goat-Ig/ peroxidase	Vector	DAB	target retrieval solution pH 6.1

Abbreviations: AP: alkaline phosphatase; DAB: 3,3’-diaminobenzidine; m:monoclonal; MVD: microvessel density p: polyclonal; TAM: tumor associated macrophage; TEM: Tie2-expressing monocyte.

### Density quantification of cellular infiltrates, angiopoietins and tumor microvascular density

To quantify TEMs, the sections were double immunostained for CD14 and TIE2 [20]. Staining of CD68 and CD163 was used to quantify TAMs or M2-polarized TAMs, respectively. Sections were stained for angiopoietin-1 and angiopoietin-2 to estimate their expression in tumor cells. After immunostaining, the whole tumor area was thoroughly inspected for the presence of antibody-positive cells. Infiltrating immune cells and angiopoietin-positive tumor cells were categorized as negative/absent in up to 5% positive cells (0–5% positive cells, score 0) and positive/present (> 5% positive cells, score 1). Subsequently, patients were divided into two groups either to be negative or positive for TEMs, CD68^+^ or CD163^+^ TAMs, and angiopoietin-1 or -2^+^ tumor cells (Table 4). TIF could be microscopically visualized in 82 out of 106 (82/106) patients.

Microvessels were identified by CD31 expression. Quantification of CD31^+^ microvessels was performed as described [30]. Briefly, the area of highest MVD, that is the vascular hot spot, was located by scanning the whole tumor section. All microvessels were counted in this spot. MVD was calculated from the number of vessels in ten random optical fields/section. MVD was categorized as low or high (MVD^low^ and MVD^high^; cut-off value: 50 microvessels/10 optical fields, Table 4).

### Statistical analysis

Survival analysis, univariate analysis and Kaplan–Meier curves were generated with the SPSS software program (Version 23.0.0.0/Year 2015). Comparison of categorical and continuous variables was performed using the Chi2-test and the Wilcoxon-test, respectively. Survival data were compared with the log-rank-test. Variables with a significant influence on survival in the univariate analysis were entered into a Cox regression analysis. A difference was considered significant for *p* < 0.05.

## Abbreviations

Ang: angiopoietin
DAMP: damage associated molecular patterns
HCC: hepatocellular carcinoma
H&E: hematoxylin and eosin
mAb: monoclonal antibody
TAM: tumor associated macrophage
TCA: tumor central area
TEM: Tie2-expressing monocytes
TIF: tumor infiltrating front
TME: tumor microenvironment
UICC: Union for International Cancer Control
VEGF: vascular endothelial growth factor
